# Supplementary material for: Identification and functional analysis of non-coding regulatory small RNA FenSr3 in Bacillus amyloliquefaciens LPB-18
Source: PeerJ. 2023 May 15;11:e15236. doi: 10.7717/peerj.15236 (PMC10194069; doi:10.7717/peerj.15236)
Supplement: Supplemental Information 4 [file peerj-11-15236-s004.zip › KO/CK-vs-T1_map/map04062.html]

KEGG PATHWAY: Chemokine signaling pathway - Reference pathway


|  |  |
| --- | --- |
| **Chemokine signaling pathway - Reference pathway** |  |

[
Pathway menu
| Organism menu
| Pathway entry
| Show description
| User data mapping
]

Inflammatory immune response requires the recruitment of leukocytes to the site of inflammation upon foreign insult. Chemokines are small chemoattractant peptides that provide directional cues for the cell trafficking and thus are vital for protective host response. In addition, chemokines regulate plethora of biological processes of hematopoietic cells to lead cellular activation, differentiation and survival.
The chemokine signal is transduced by chemokine receptors (G-protein coupled receptors) expressed on the immune cells. After receptor activation, the alpha- and beta-gamma-subunits of G protein dissociate to activate diverse downstream pathways resulting in cellular polarization and actin reorganization. Various members of small GTPases are involved in this process. Induction of nitric oxide and production of reactive oxygen species are as well regulated by chemokine signal via calcium mobilization and diacylglycerol production.

|  |  |  |
| --- | --- | --- |
| Reference pathway | 184% 150% 122% 100% 82% 67% 55% | 图片下载 |
